# Optimization of seminal extracellular vesicle isolation protocols for cancer biomarker analysis using proteomic profiling

**DOI:** 10.1002/1878-0261.70345

**Published:** 2026-10-22

**Authors:** Elen Telumyan, Beatriz Martín‐Gracia, Eva Bassols‐Citores, Krizia Sagini, Maria K. Andersen, Tuula A. Nyman, May‐Britt Tessem, Morten B. Rye, Alicia Llorente

**Affiliations:** ^1^ Department of Circulation and Medical Imaging NTNU Trondheim Norway; ^2^ Department of Molecular Cell Biology, Institute for Cancer Research Oslo University Hospital‐The Norwegian Radium Hospital Oslo Norway; ^3^ Centre for Cancer Cell Reprogramming, Faculty of Medicine University of Oslo Norway; ^4^ Department of Immunology University of Oslo and Oslo University Hospital Norway; ^5^ Clinic of Surgery, St. Olavs Hospital Trondheim University Hospital Norway; ^6^ Department of Clinical and Molecular Medicine NTNU Trondheim Norway

**Keywords:** EV isolation, EV‐based biomarkers, liquid biopsies, prostate cancer, proteomics, seminal small EVs

## Abstract

Seminal extracellular vesicles (EVs) hold promise as a source of biomarkers for urogenital cancers. In this study, small seminal EVs were isolated from healthy donors by size‐exclusion chromatography (SEC) and three ultracentrifugation (UC) protocols, with or without filtration. EV samples prepared by UC had a higher abundance of typical EV‐related molecules such as CD9, CD81, and Alix, compared to SEC preparations. However, ribosomal and ER‐related proteins were more enriched in SEC samples. Furthermore, the level of several nonvesicular proteins and/or EV‐corona proteins was higher in SEC samples. The addition of a filtration step in the UC protocols also reduced the abundance of these proteins. Finally, we found that the isolation protocol affected the presence of prostate cancer‐specific proteins in the EV samples, with membrane proteins such as FOLH1, TMPRSS2, and PSCA enriched in samples prepared by UC. In conclusion, our results suggest that SEC and UC enrich for different EV populations, which should be considered when analyzing cancer biomarkers. Moreover, UC samples contained fewer nonvesicular proteins and/or EV‐corona proteins, which may enable a more precise detection of EV‐based cancer biomarkers.

## Graphical abstract

This paper shows that size‐exclusion chromatography and sequential centrifugation applied to seminal plasma enrich for different populations of extracellular vesicles. This may significantly affect the detection and analysis of extracellular vesicle‐associated cancer biomarkers. Therefore, the choice of protocol for extracellular vesicle isolation is an important consideration in biomarker research. Figure created with biorender. Telumyan, E. (2026). Telumyan, E. (2026) https://BioRender.com/95v2qle (agreement number SJ2A8CTEAZ).

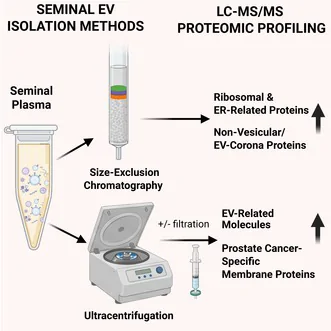

**Abbreviations**
EVs: extracellular vesicles
FDR: false discovery rate
FOLH1: glutamate carboxypeptidase 2
MS: mass spectrometry
PAP: prostatic acid phosphatase
PCa: prostate cancer
PSA: prostate‐specific antigen
PSCA: prostate stem cell antigen
PSMA: prostate‐specific membrane antigen
SEC: size‐exclusion chromatography
SEM: semenogelin
STEAP4: six‐transmembrane epithelial antigen of prostate 4
TGM4: protein‐glutamine gamma‐glutamyl transferase 4
TMPRSS2: transmembrane serine protease 2
UC: ultracentrifugation

## 1 Introduction

Over the past decade, considerable attention has been devoted to the discovery of novel cancer biomarkers in liquid biopsies. This minimally invasive approach provides substantial advantages to clinical care, avoiding patient discomfort and reducing clinical workload [1, 2]. Semen is an insightful biofluid for noninvasive disease assessment. It is mainly composed of secretions from several glands in the male urogenital tract such as the seminal vesicles (~ 65%), the prostate (~ 25%), the epididymis, the testicles (together ~ 10%), and the bulbourethral glands (~ 1%) [3]. It therefore may contain valuable biomarkers that can reflect the pathophysiology of several urogenital diseases, including prostate cancer (PCa), testis cancer, but also benign prostatic hyperplasia, prostatitis, and male infertility.

Extracellular vesicles (EVs) are a key component of semen that is promising in the search for potential biomarkers [4]. Seminal EVs are membrane‐bound vesicles ranging from 40 to 500 nm in diameter that are secreted from multiple parts of the male reproductive tract, such as the seminal vesicles, the prostate, the epididymis, and the testicles [5, 6, 7, 8, 9]. The lipid bilayer of EVs act as a protective shield for their molecular cargo, including proteins, nucleic acids, lipids, and metabolites [10, 11, 12, 13]. This cargo reflects the pathophysiological status of the male urogenital system, making seminal EVs a potentially powerful diagnostic tool.

The first proteomic profiling of seminal EVs using mass spectrometry (MS) was conducted by Utleg et al. in 2003 [14]. Among the 139 proteins identified, there were several prostate‐related proteins such as prostate‐specific antigen (PSA, KLK3), prostatic acid phosphatase (PAP), and transmembrane serine protease 2 (TMPRSS2). Proteins originating from the seminal vesicles were also found, such as the semenogelin (SEMG) proteins SEMG1 and SEMG2. These proteins are the most abundant proteins in seminal plasma [15], making up to 40–50% of the total protein content. They actively take part in semen coagulation after ejaculation and are subsequently cleaved by PSA, leading to the liquefaction of the seminal coagulum [16, 17, 18]. Some years later, Poliakov et al. [19] identified 440 proteins in seminal EVs and confirmed the presence of prostate‐, seminal‐ and epididymal proteins, supporting the idea of the multi‐organ origin of seminal EVs. This has also been confirmed by several proteomic studies of human seminal EVs later, including a recent study that identified over 5000 proteins in seminal EVs [7, 20, 21, 22, 23]. In addition, in pooled batches of seminal plasma from three donors, Zhang et al. [23] identified two distinct populations of seminal small EVs of approximately 100 and 50 nm by sucrose gradient centrifugation. In addition, it has also been found that in pig seminal plasma, there are several proteomic differences between large and small EVs (pelleted or not at 20 000× ***g***, respectively) [24]. In both cases, a GO analysis suggested that the two populations may have different biological functions and biogenesis mechanisms.

Considering the noninvasive nature of semen collection and the functional and pathological relevance of seminal EVs, optimizing the existing isolation methods is crucial for translating seminal EV‐based biomarkers into diagnostic tests. The choice of EV isolation method is typically guided by four key factors: EV yield, EV purity, hands‐on laboratory work, and technical reproducibility [25, 26]. Several techniques have been used to obtain seminal EVs, such as size‐exclusion chromatography (SEC), differential centrifugation, sucrose gradient centrifugation, filtration, and combinations of these methods [23, 24, 27, 28, 29, 30, 31]. These methods have not often been systematically compared in the same study, but analysis of separate studies using different methods indicates that the EV molecular profile largely depends on the EV separation method [7]. These differences may be due to several reasons, such as differences in contaminant removal, EV corona preservation, or isolation of specific seminal EV populations. Interestingly, it has recently been shown that the EV corona can affect the accessibility of EV surface proteins [32].

In this study, we focused on small EVs, which generally refer to EVs that are lower than 200 nm in diameter [33]. Cells, cell debris, and large EVs were removed first from the semen samples by sequential centrifugation at 1600× ***g*** and 16 000× ***g***. Then, small EVs were isolated by SEC or three ultracentrifugation (UC)‐based protocols that differed only by the inclusion of a 200‐nm filtration step. Our results offer valuable insights into the performance of the different techniques. Additionally, proteomic analysis supports the heterogeneity of seminal EVs with respect to both organ origin and biogenesis.

## 2 Materials and methods

### 2.1 Sample collection

Semen samples were collected from five healthy donors (aged 20–39) in 2024 at St. Olavs Hospital, Trondheim, Norway. The study methodologies conformed to the standards set by the Declaration of Helsinki. The collection was approved by the Norwegian Regional Committees for Medical and Health Research Ethics (REK Approval 2012/335), and participants provided written informed consent. Comprehensive instructions for sample collection were provided, ensuring an easy‐to‐follow approach for home collection of semen through masturbation.

### 2.2 Clearing of seminal samples

After collection, samples were kept at RT (approximately 20 °C) for up to 2 h to allow for complete liquification. This period is critical, as it ensures that semen transitions from a gel‐like state to a liquid form, which is essential for further preparation and analysis of seminal plasma. Following liquefaction, semen samples were precleared to remove sperm cells, cellular debris, and large EVs. The first centrifugation was conducted at 1600× ***g*** for 10 min at 4 °C to pellet the spermatozoa, other cells, and cell debris. The supernatant was then carefully collected and centrifuged at 16 000× ***g*** for 15 min at 4 °C to pellet any remaining cellular components and large EVs. Finally, the supernatant was recovered, split into 1‐mL aliquots, and stored at −80 °C until further use (Fig. 1).

**Fig. 1:**
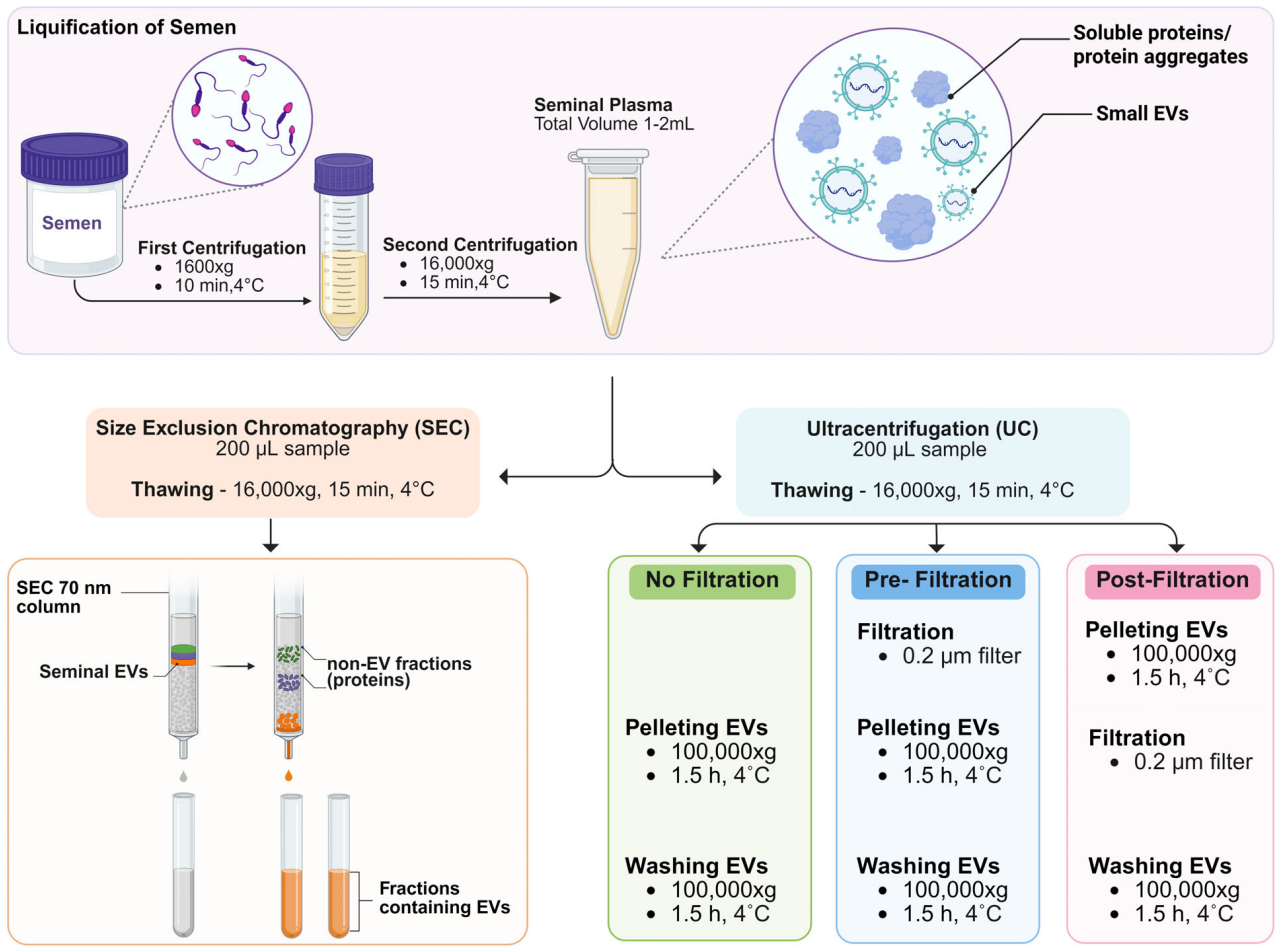
Schematic diagram of the procedures used in this study to isolate small seminal EVs. Freshly collected semen samples from five donors were liquefied at room temperature for up to 2 h. This was followed by centrifugation at low speeds to remove cells, cell debris, and large EVs. The samples were then frozen until further use. For isolation of small EVs, the samples were thawed and centrifuged to remove potential precipitates resulting from the freeze–thaw cycle. EVs were then isolated by UC or SEC, as indicated. After isolation, EVs were analyzed by dynamic light scattering, electron microscopy, western blot, bicinchoninic acid protein assay, and mass spectrometry. Due to limited sample availability, not all EV samples were included in each analysis. The number of donors and technical replicates for each specific EV analysis is indicated in the corresponding figure legends. EVs, extracellular vesicles; SEC, size‐exclusion chromatography; UC, ultracentrifugation. Figure created with biorender. Telumyan, E. (2026) https://BioRender.com/h4jjebj (agreement number TL2A61TS02).

### 2.3 Isolation of seminal EVs from precleared seminal plasma by ultracentrifugation

For EV isolation, the precleared seminal plasma samples were thawed and centrifuged again at 16 000× ***g*** for 15 min at 4 °C to remove potential precipitates resulting from the freeze–thaw cycle. Afterward, the supernatant (200 μL) was collected and subjected to ultracentrifugation at 100 000× ***g*** for 1.5 h at 4 °C in an MLA‐80 rotor using a MAX‐XP ultracentrifuge (Beckman Coulter Life Sciences, Indianapolis, IN, USA) (Fig. 1). The resulting pellets were washed with filtered PBS (0.2 μm filter) and pelleted at 100 000× ***g*** for 1.5 h at 4 °C. Additionally, a 0.2 μm filtration step with Acrodisc Syringe Filters with Supor Membrane (polyethersulfone) (Cytiva, VWR International, Norway) was introduced for some samples. The filtering process was applied either before (prefiltration) or after (postfiltration) pelleting the EVs. Ultimately, the EV preparations were resuspended in 200 μL PBS for further analysis (Fig. 1).

### 2.4 Isolation of seminal EVs from precleared semen by size exclusion chromatography

The precleared seminal samples (200 μL) were thawed and centrifuged at 16 000× ***g*** for 15 min at 4 °C to remove potential precipitates resulting from the freeze–thaw cycle (Fig. 1). Meanwhile, the SEC columns (qEV single, 70 nm pore size, IZON, Lyon, France) were pre‐washed with filtered PBS. The samples (170 μL) were then carefully overlaid onto the columns, and the EVs were eluted with filtered PBS using an automatic fraction collector (IZON, Lyon, France), yielding 12 fractions of 200 μL.

### 2.5 Total protein measurements

The total amount of protein in the samples was measured with the bicinchoninic acid (BCA) assay kit (Thermo Fisher Scientific, Waltham, MA, USA) following the manufacturer's instructions for the enhanced protocol. Bovine serum albumin was used as a standard protein. The absorbance was measured at 562 nm using a Biotek Synergy 2 Plate Reader (Agilent Technologies, Santa Clara, CA, USA). To study the statistical differences between all conditions, one‐way ANOVA with Dunnett's test was used. For the comparisons within UC protocols, the nonfiltered samples were used as the reference group, as they consistently showed the highest values. In the comparison between the SEC and UC methods, the SEC served as the reference, following the same rationale.

### 2.6 SDS/PAGE and western blot

Seminal EVs were dissolved in lysis buffer (50 mm Tris–HCl, 300 mm NaCl, 1 mm EDTA, 0.5% Triton X‐100, pH 7.4) containing an EDTA‐free protease inhibitor mixture and then Laemmli sample buffer (Bio‐Rad, Hercules, CA, USA) was added. For analysis of the SEC fractions, the same volume of each fraction was loaded into gels because the fractions have different concentrations of EVs. For comparison of EVs isolated by different protocols, the samples were normalized by an equal volume of seminal fluid from which the EVs were isolated. This is because the different EV isolation protocols may yield different EV concentrations and/or co‐isolation of non‐EV proteins. Samples were separated by SDS/PAGE (4–20%) using the Mini‐Protean system (Bio‐Rad, Hercules, CA, USA). Proteins were transferred onto a PVDF membrane, which was then blocked with 5% skim milk powder in PBS‐T (0.1% Tween‐20) for 1 h at RT. After blocking, the membrane was incubated overnight at 4 °C with primary antibodies, followed by washing in PBS‐T. Secondary antibodies conjugated with horseradish peroxidase (HRP) were diluted in 5% skim milk powder in PBS‐T and incubated with the membrane for 1 h at RT under shaking. The following HRP substrates were used depending on the required sensitivity: HRP substrate for chemiluminescent Westerns (Azure Biosystems, Dublin, California, USA), SuperSignal West Dura Extended Duration Substrate (Thermo Fisher Scientific, Waltham, MA, USA), or SuperSignal West Femto Maximum Sensitivity Substrate (Thermo Fisher Scientific). Finally, the signals were visualized with a ChemiDoc Imaging System (Bio‐Rad). The following primary antibodies were used: anti‐CD9 (ab263019; Abcam, Cambridge, UK), anti‐CD63 (H5C6, DSHB; Iowa City, IA, USA), and anti‐PSMA, CD81, and SEMG1 (sc‐365 939; Santa Cruz Biotechnology, CA, USA). Densitometric analysis of western blot bands was performed using the imagej software. Relative signal intensities were normalized to the unfiltered samples, which served as the reference group (the highest in this dataset). Statistical comparisons of marker expression (CD9 and CD81) between isolation methods were conducted using one‐way ANOVA, followed by Dunnett's post hoc test.

### 2.7 Electron microscopy

For negative staining, seminal EVs were applied to formvar/carbon‐coated grids and incubated for 5 min. The grids were rinsed twice with PBS, followed by four washes with MilliQ water and subsequently stained with 4% uranyl acetate for 3 min. After the final wash, the grids were dried for 10 min. Samples were then visualized using a JEOL‐JEM 1230 at 80 kV. Images were captured using a Morada digital camera and further processed with imagej.

### 2.8 Dynamic light scattering

The size and count rate of seminal EV preparations was measured by dynamic light scattering using a Zetasizer Nano ZS (Malvern Instruments, Malvern, UK). This technique is based on the detection of scattered light caused by the Brownian motion of particles in a liquid medium.

### 2.9 Proteomic analysis

Samples from two donors were used for the proteomic analysis. Each donor sample was divided into aliquots, and EVs were isolated independently from two aliquots per method and donor. This resulted in four EV samples being analyzed for each method, representing both biological and technical variability. From each of the 14 seminal EV preparation, 1 μg of protein was precipitated on hydroxyl beads as previously described [34]. The precipitated proteins were dissolved in 50 mm ammonium bicarbonate, reduced, alkylated, and digested with trypsin (1 : 50 enzyme : protein ratio; Promega, Madison, WI, USA) at 37 °C overnight. The resulting tryptic peptides were purified using homemade C18 stage tips and analyzed by a nanoElute UHPLC coupled with a timsTOF PRO2 mass spectrometer (Bruker Daltonics, Bremen, Germany) through a Captive Spray ion source using a 60‐min separation gradient (0–35% acetonitrile). The MS instrument was operated in DIA‐PASEF mode. Mass spectra for MS were recorded between m/z 100 and 1700. Ion mobility resolution was set to 0.85–1.30 V·s·cm^−1^ over a ramp time of 100 ms. The MS/MS mass range was limited to m/z 475–1000 and ion mobility resolution to 0.85–1.30 V s·cm^−1^ to exclude singly changed ions. The estimated cycle time was 0.95 s with eight cycles using DIA windows of 25 Da. Collisional energy was ramped from 20 eV at 0.60 V s·cm^−1^ to 59 eV at 1.60 V s·cm^−1^.

Raw data was analyzed using DIA‐NN (v1.9.2) for protein identification and label‐free quantification, employing an *in silico* human FASTA library from Uniprot and a contaminant database from maxquant. Carbamidomethyl (C) was set as a fixed modification. Trypsin without proline restriction cleavage option was used, with one allowed miscleavage and peptide length range set to 7–30 amino acids. The mass accuracy was set to 15.0 ppm (as recommended by the DIA‐NN software manual for data generated on timsTOF). The precursor false discovery rate (FDR) allowed was 0.01 (1%), and the same value was used for protein FDR. DIA‐NN uses a decoy database to estimate the protein *q*‐values. Proteins were considered uniquely identified in the sample if one proteotypic peptide was found (DIA‐NN default settings). If only peptides matching to more than one protein were found, a protein Group was reported. The MS proteomics data have been deposited to the ProteomeXchange Consortium via the PRIDE [35] partner repository with the dataset identifier PXD069636.

The normalized LFQ intensities from DIA‐NN were processed and analyzed in R. Venn diagrams were performed using the R package ggVenn. To detect differences in protein abundances among the samples, only proteins identified in all 14 samples were considered. Ratios between the abundance of the identified proteins in the different conditions and a specific control, SEC in one analysis and UC no filtration in the other, were calculated. For each analysis, four ratios and their *P*‐values were calculated based on the four samples available for each condition. Proteins showing increased or decreased abundance by a ratio larger than 1.5 compared to the control in all the four samples were selected. The four ratios were used to calculate the mean ratio and standard deviation. The heatmaps were generated using the R package ComplexHeatmap [36], using log_10_‐transformed ratios for better visualization.

## 3 Results

### 3.1 Analysis of seminal small EV preparations obtained by SEC

Semen was collected from healthy men, and sperm cells, cellular debris, and large EVs were removed by centrifugation at 1600× ***g*** and then at 16 000× ***g*** (Fig. 1). The remaining EVs in the samples were considered to be small EVs [33], and SEC was used to separate them from non‐EV soluble seminal proteins [37, 38]. To confirm the efficiency of the method, 170 μL of cleared seminal plasma was placed on top of a 70 nm SEC column, and the total protein amount per fraction was quantified using the BCA assay. The highest amount of protein was recovered in Fractions 9–12 (Fig. 2A) (approximately 85%), suggesting that mostly soluble proteins contribute to the total amount of protein in seminal plasma. Then, dynamic light scattering was used to compare the signal strength (scattering intensity) of the fractions measured as count rate. A higher count rate usually indicates higher particle concentration, larger particles, or both. As shown in Fig. S1, the count rate was highest in Fractions 3 and 4 and very low in the latter fractions, suggesting that Fractions 3 and 4 could contain an EV‐enriched solution. The hydrodynamic diameter of the particles in Fraction 3 and 4 was 152.0 ± 4.7 (*n* = 3) and 152.3 ± 3.8 (*n* = 3), respectively.

**Fig. 2:**
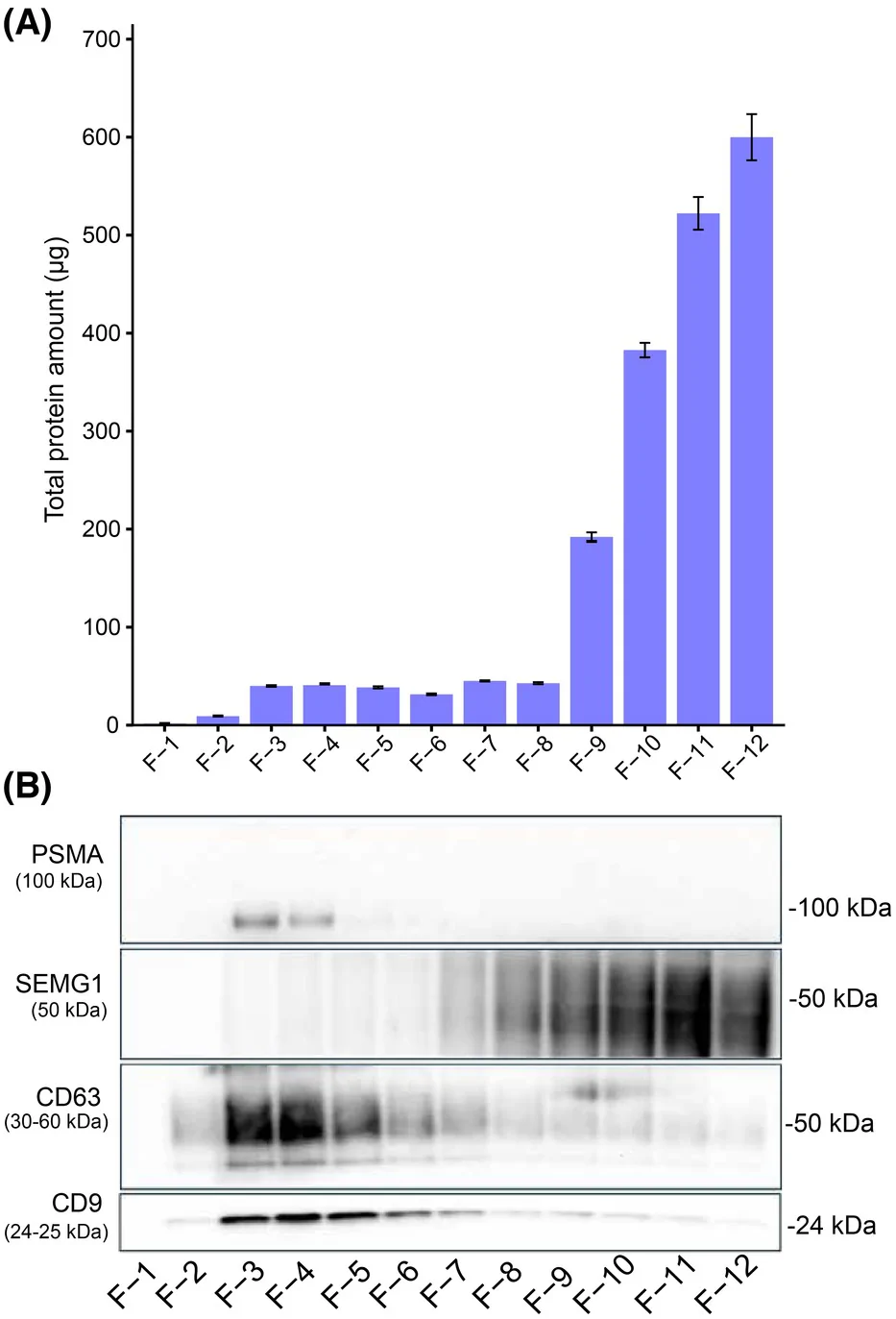
Analysis of cleared seminal fluid by SEC. (A) Protein quantification of SEC fractions by the bicinchoninic acid protein assay. The figure shows the mean and standard deviation between the duplicates of a representative donor; similar results were consistently observed across three donors. (B) Western blot analysis of SEC fractions using EV‐related markers (CD63, CD9), a prostate‐specific protein (PSMA, prostate‐specific membrane antigen), and a seminal vesicle protein (SEMG1, semenogelin 1). The same volume of each fraction was loaded into the gel. The figure shows the result of a representative donor; similar results were consistently observed across three donors. EV, extracellular vesicle; F, fraction; SEC, size‐exclusion chromatography.

To determine whether Fractions 3 and 4 contain EVs, the presence of the EV‐related proteins CD63 and CD9 in similar volumes of the SEC fractions was analyzed by western blot. The signals of these proteins were highest in Fractions 3 and 4, suggesting the presence of EVs in these fractions (Fig. 2B). Prostate‐specific membrane antigen (PSMA), a membrane‐bound prostate‐related protein that has previously been found in EVs [39, 40], was also detected in these fractions, thereby supporting the presence of EVs of prostatic origin in the samples. The presence of SEMG1, a protein secreted by the seminal vesicles and the most abundant protein in seminal plasma, was also investigated. As shown in Fig. 2B, SEMG1 was mainly associated with the latest SEC fractions, suggesting that most of it is not associated with EVs. However, a small fraction of SEMG was found in Fraction 3–4, which may be due to the formation of large SEMG1 aggregates and/or to the presence of SEMG in the EVs or EV corona [41]. Based on these results, Fractions 3 and 4 were considered to contain EVs and were commonly selected and pooled for downstream analysis.

### 3.2 Comparative analysis of SEC and UC for seminal small EV isolation

Precleared seminal fluid from each donor was divided into seven aliquots to allow direct comparison of the four EV isolation protocols (Fig. 1), being the three UC‐based protocols performed in duplicate and the SEC protocol as a single sample. In the UC‐based protocols, small EVs were pelleted by ultracentrifugation at 100 000× ***g***, adding or not adding a 200‐nm filtration step before or after EV pelleting (UC No filtration, UC Prefiltration, and UC Postfiltration). The filtration step was introduced to study whether it may provide EV preparations with reduced co‐isolation of large protein complexes, as it has been shown for urinary EVs and uromodulin [42]. The protein content of EV samples prepared from similar volumes of cleared seminal sample was then analyzed using the BCA assay. As shown in Fig. 3A, the SEC‐EV samples consistently showed higher protein levels compared to UC preparations. However, this does not necessarily mean that there are more EVs in the samples. It could also be due to SEC being less efficient at removing nonvesicular proteins from the EV fractions than UC. To clarify this, the EV‐related proteins CD81 and CD9 were analyzed by western blot. As shown in Fig. 3B–D, the signals of CD9 and CD81 were lower in SEC‐EVs compared with the UC‐EVs processed without filtration. This result suggests that SEC does not isolate more EVs than UC, or at least not EVs containing CD9 and CD81. Considering the effect of the 200‐nm filtration step in the UC protocols, the amount of protein was lower in the filtrated samples compared to the nonfiltrated samples, especially in the postfiltered samples (Fig. 3A). Moreover, as shown in Fig. 3B–D, the signals of CD9 and CD81 were lower in the filtrated UC‐EV samples, especially in the postfiltration samples, compared to the filtrated samples. This result suggests that even if the filtration step may eventually retain large nonvesicular protein complexes and reduce their co‐isolation with EVs, it does so at the cost of a lower EV yield. Finally, the samples were observed by electron microscopy to confirm the presence of EVs. As shown in Fig. 3E, all the samples contained EVs, and they showed similar morphology.

**Fig. 3:**
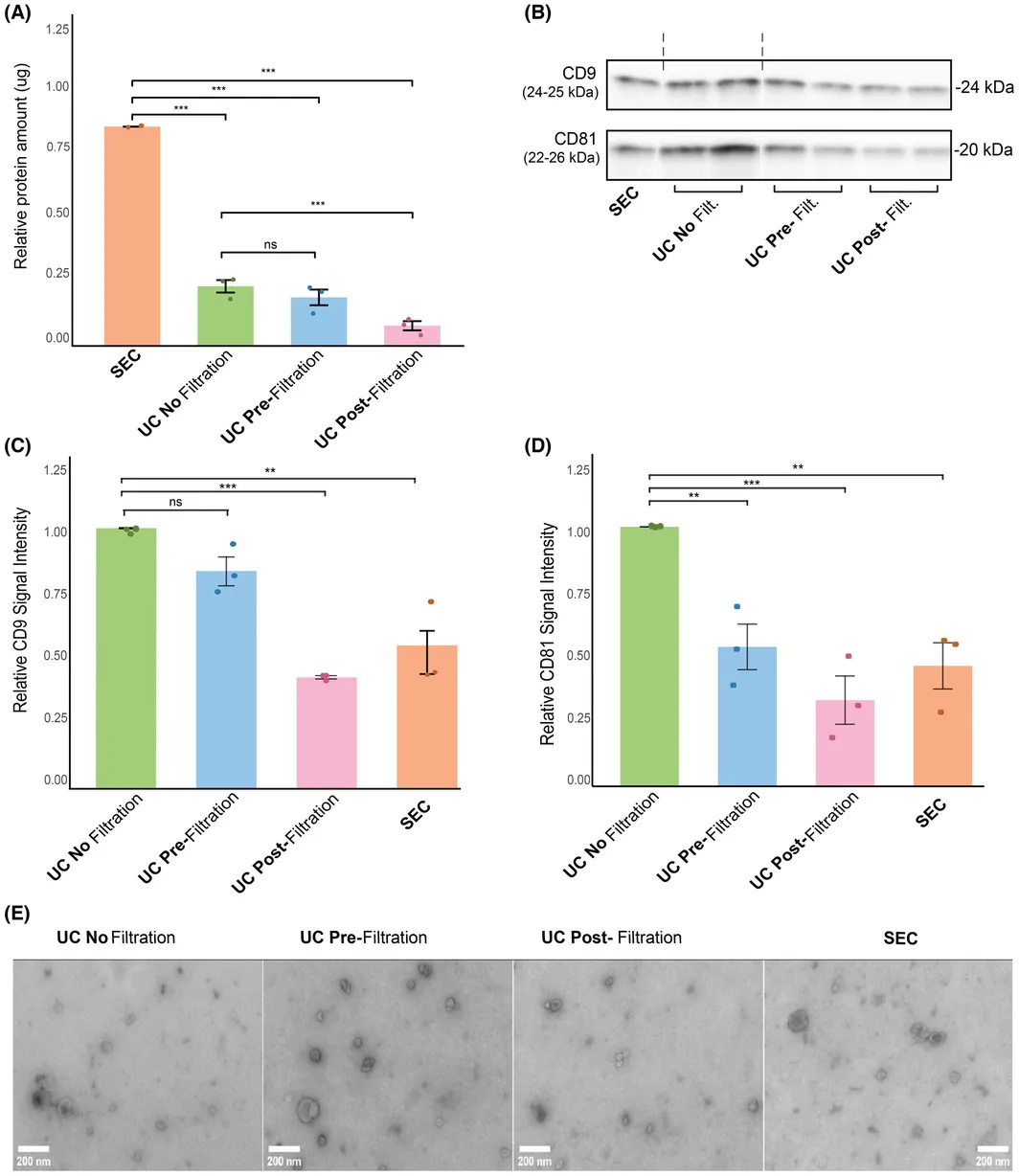
Comparison of seminal small EVs isolated by SEC and UC. (A) Total protein amount in the EV fraction obtained using the specified protocols, as measured by the bicinchoninic acid assay. (B) EV samples obtained from similar volumes of seminal plasma using the specified isolation methods were analyzed by western blot using antibodies against the EV‐related proteins CD9 and CD81. A representative experiment from one of three donors is shown. The UC protocols were performed in duplicate. The picture of the gel was cut (vertical lines) to maintain a consistent order of the protocols throughout the manuscript. (C) Quantification of the western blot CD9 signals in small EV samples prepared using the indicated protocols. (D) Quantification of the western blot CD81 signals in small EV samples prepared using the indicated protocols. (E) Representative electron microscopy images of small seminal EVs samples from one donor prepared using the indicated protocols. Scale bar: 200 nm. Error bars represent standard deviation from three donors (*n* = 3). The UC protocols were performed in duplicate for each donor. Statistical significance was assessed using one‐way ANOVA followed by Dunnett's *post hoc* test. Asterisks (*) denote significance levels between compared samples: *P* < 0.01 (**), and *P* < 0.001 (***). EVs, extracellular vesicles; Filt, filtration; ns, not significant; SEC, size‐exclusion chromatography; UC, ultracentrifugation.

### 3.3 Proteomic analysis of seminal small EVs prepared by SEC and UC

To gain a deeper insight into the protein composition of seminal small EV samples prepared by UC and SEC, we performed an MS‐based proteomic analysis in samples from two healthy donors (14 samples in total). A total of 1 μg of protein from each sample was analyzed. This resulted in the detection of a mean of 3346 proteins per sample (Fig. 4A). All the identified proteins can be found in File S1, Table S1 and in the PRIDE [35] partner repository with the dataset identifier PXD069636. Over 70% of the proteins were common among the two donor samples when isolated by the same UC protocol (Fig. 4B), and the protein overlap between the donors when using SEC was of 84% (Fig. 4C). Principal component analysis showed that the three UC‐based protocols in each donor grouped closely together, whereas the SEC‐EV samples behave differently (Fig. S2). Next, we selected the 100 most abundant proteins per sample and, as shown in Fig. 5, 54.3% of these proteins were shared across all the samples. Among these proteins, several proteins normally found in EVs such as tetraspanins (CD9, CD81, tetraspanin 1), tsg101, syntenin, Alix, annexins, Rabs, and heat shock proteins were identified (Fig. 5). The lysosomal membrane proteins LAMP1 and LAMP2, and the plasma membrane enzymes CD13 (ANEP), CD26 (DPP4), and CD10 (MME) were also abundant in all the EV samples (Fig. 5). In addition, several prostate‐associated proteins, such as prostate stem cell antigen (PSCA), PAP, protein‐glutamine gamma‐glutamyl transferase 4 (TGM4), glutamate carboxypeptidase 2 (FOLH1), six‐Transmembrane Epithelial Antigen of Prostate 4 (STEAP4), TMPRSS2, Rab3 and beta‐microseminoprotein (Fig. 5), were abundant in the samples. Finally, SEMG1 and SEMG2, as well as mucin 5B and mucin 6, were also found among the abundant common proteins in seminal EV samples (Fig. 5). These proteins, as well as other secreted proteins such as KLK2, KLK3, clusterin, fibronectin and pancreatic glycoprotein 2 (Fig. 5), may be part of the EV corona and/or form aggregates that have been copurified with EVs. Of note, the latter proteins were found only in EVs prepared by SEC and/or UC postfiltration. Finally, other proteins typically found in EVs such as CD63 and other tetraspanins (TSPAN6, TSPAN8) and ESCRT proteins (VPS28, VPS37C) were found in the top100 of all the three UC‐based protocols.

**Fig. 4:**
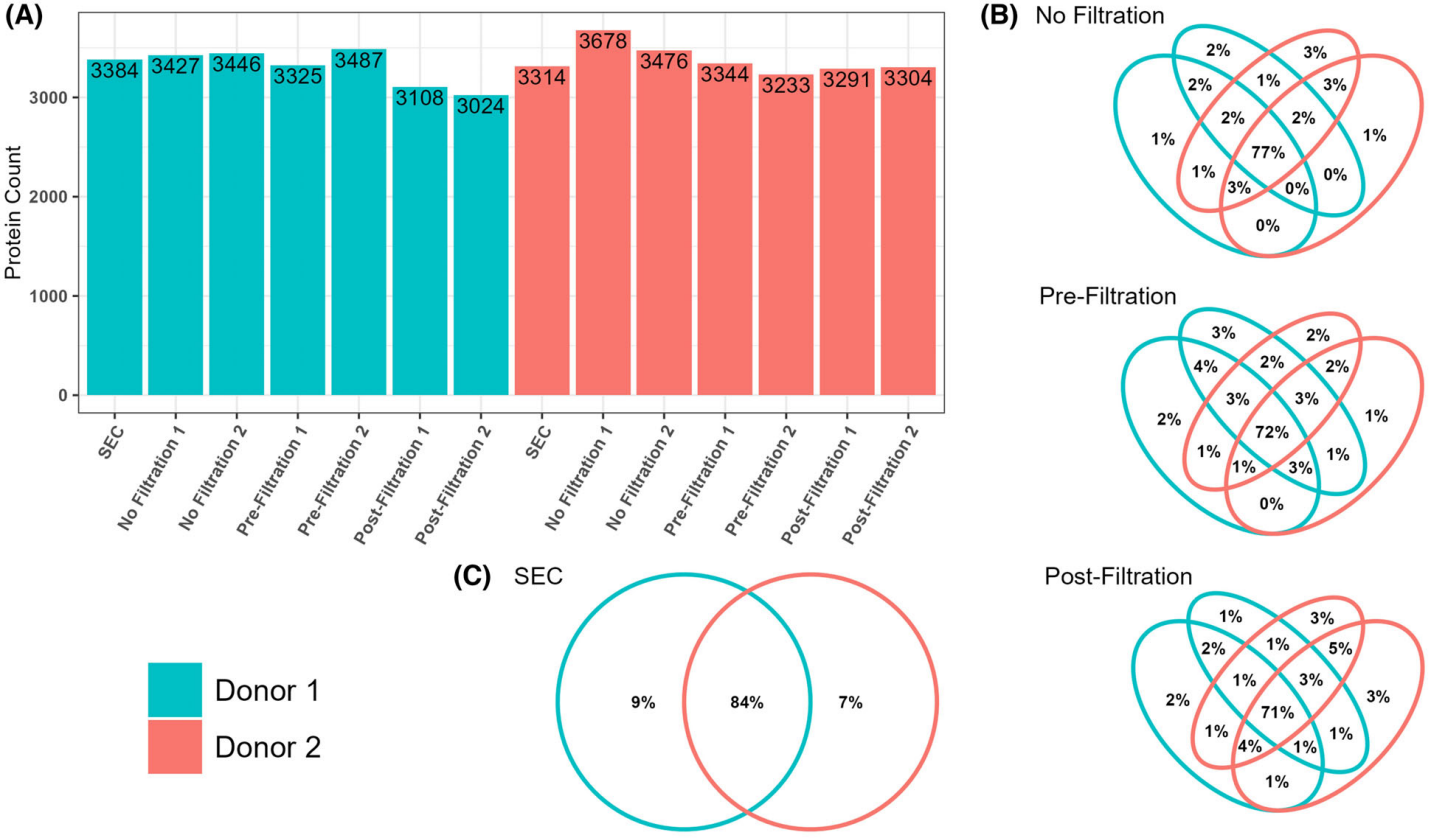
Proteins identified by mass spectrometry in seminal small EVs prepared by SEC and by UC. In total 14 samples of small EVs were analyzed: seven samples per donor (1 sample prepared by SEC, and the UC no filtration, UC prefiltration and UC postfiltration samples prepared in duplicate). (A) Number of detected proteins per sample. (B) Venn diagrams showing the percentage of shared proteins across each UC protocol. In each diagram four EV samples prepared with the same protocol are included, corresponding to duplicates from two donors. (C) Venn diagram showing the percentage of shared proteins in small EV samples prepared by SEC from two donors. EVs, extracellular vesicles; SEC, size‐exclusion chromatography; UC, ultracentrifugation.

**Fig. 5:**
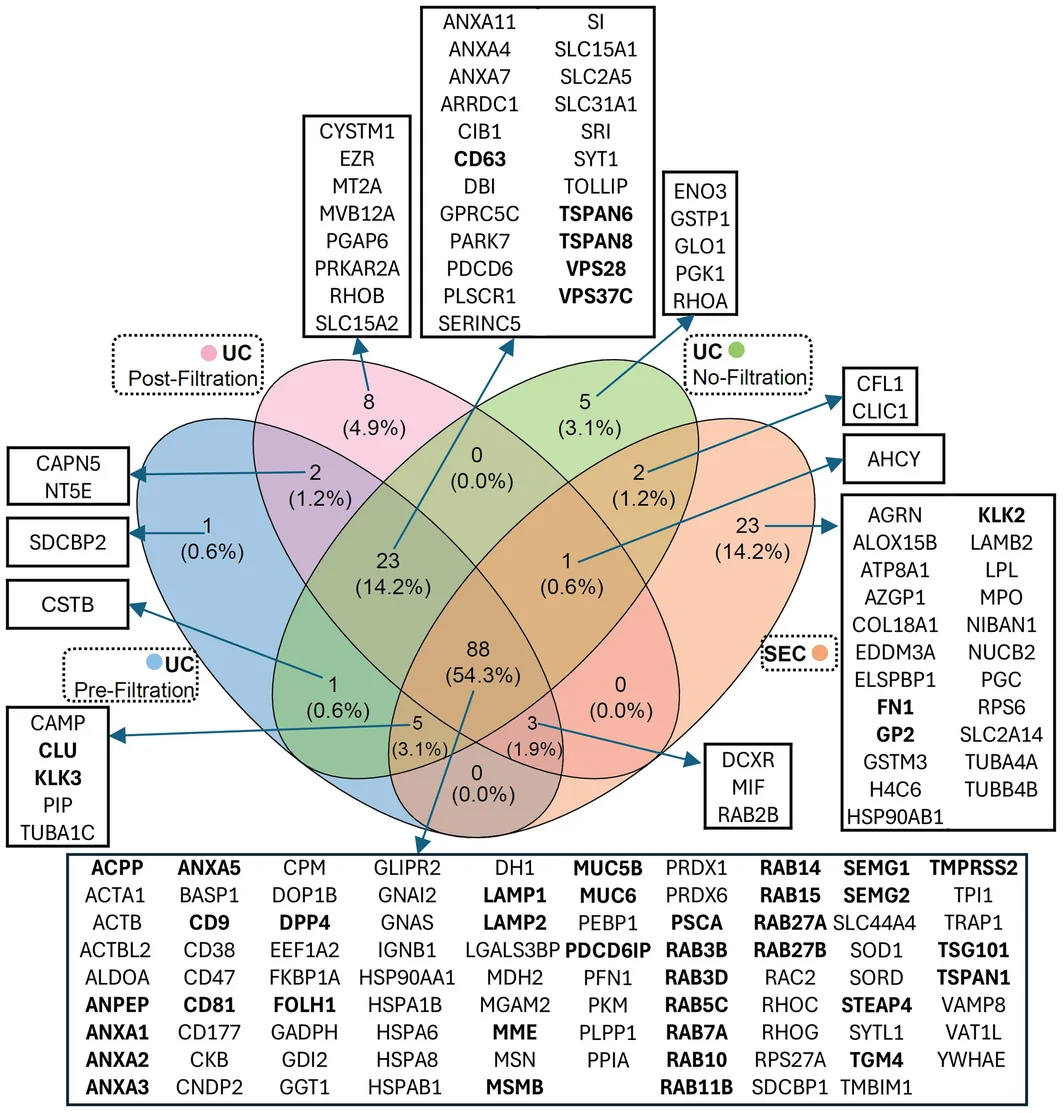
Venn diagram of the most abundant proteins in seminal small EVs prepared by different protocols. The Venn diagram shows the overlap of the 100 most abundant proteins identified by mass spectrometry in EVs prepared by the specified protocols. The top proteins for each protocol were obtained by combining the replicates across both donors. The gene names are indicated in alphabetical order, and the genes mentioned in the text are in bold. EVs, extracellular vesicles; SEC, size‐exclusion chromatography; UC, ultracentrifugation.

To further understand the possible differences in protein composition between EVs prepared by SEC and by UC, ratios and their *P*‐values between the abundance of proteins in UC‐EV samples versus SEC‐EV samples were calculated. Only proteins identified in all 14 samples were included in the analysis. This showed that 238 proteins were more abundant and 383 were less abundant (fold change 1.5) in seminal EVs prepared by SEC compared to UC no filtration (File S1, Table S2). Several protein groups/families were decreased in the UC‐EVs compared to the SEC‐EVs, including ribosomal proteins, endoplasmic reticulum (ER)–Golgi‐related proteins, secreted proteins, and lysosomal enzymes (Fig. 6). In contrast, proteins commonly associated with small EVs, such as tetraspanins, ESCRT proteins, flotillins, Rabs, and annexins, were enriched in seminal EVs prepared by UC (Fig. 6). Other proteins enriched in these EVs were proteasomal proteins, SNARE proteins, sortin nexins, and solute carrier family members (Fig. 6). These results suggest that the SEC‐EV and UC‐EV preparations do not contain identical EV populations. This is in agreement with previous studies that identified two different EV populations when small EVs from seminal fluid were prepared by sucrose gradient [23].

**Fig. 6:**
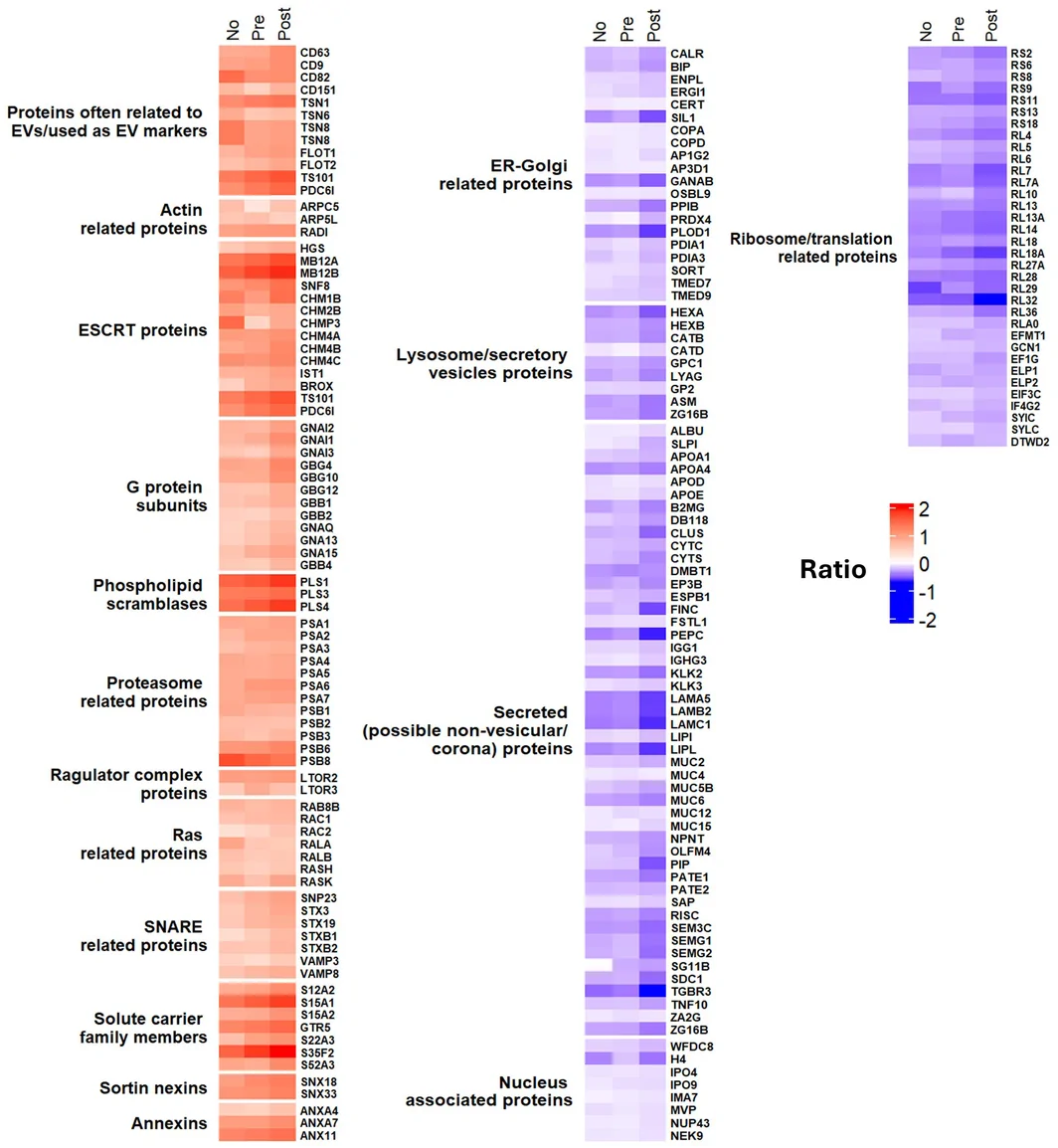
Specific groups/families of proteins differentially abundant in seminal small EVs prepared by UC compared to SEC. The heatmaps show the protein abundance ratio between the three different UC protocols and SEC. For this analysis, four ratios were calculated based on the four samples available for each UC protocol. Proteins showing decreased (blue) or increased (red) abundance by a ratio larger than 1.5 compared to SEC in all four samples were selected. The four ratios were used to calculate the mean ratio. For ease of visualization, the color scale shows the log_10_‐transformed ratios. The heatmaps include proteins whose abundance was changed in any of the three UC protocols compared to SEC. Only proteins identified in all the samples were considered. Order of columns from left to right: No, UC No‐filtration vs SEC; Pre, UC Prefiltration vs SEC; Post, UC Postfiltration vs SEC. EVs, extracellular vesicles; ESCRT, endosomal sorting complex required for transport; ER, endoplasmic reticulum; G proteins, GTP‐binding proteins; Ras, rat sarcoma; SEC, size‐exclusion chromatography; SNARE, soluble N‐ethylmaleimide‐sensitive factor attachment protein receptor; UC, ultracentrifugation.

SEMG1 and SEMG2 were among the most abundant proteins detected by MS in seminal small EV preparations, constituting approximately 11% and 8% of the total signal in SEC‐EVs from Donor 1 and 2, respectively (File S1, Table 1). SEMG1 and SEMG2 were also abundant in EVs prepared by UC, but to a lesser extent (Fig. 7A). The level of SEMG1 and SEMG2 in EVs isolated by UC was approximately 70% lower compared to EVs isolated by SEC, and this reduction was even more pronounced in the EV samples prepared using the UC post‐filtration protocol (Fig. 7A). Our experiments do not allow us to determine whether SEMG is truly found in EVs (as an intrinsic or corona protein), or whether it just co‐isolates with EVs and can be considered a contaminant [41]. This may also be the case for other proteins found in the EV preparations such as albumin, apolipoproteins, beta‐2‐microglobulin, clusterin, fibronectin, nephronectin, gastricsin, immunoglobulins, mucins, and antileukoproteinase (Fig. 6). However, based on SEC experiments (Fig. 2), it is evident that, at least for SEMG, only a minor proportion of the total protein is present in the EV‐containing fractions.

**Fig. 7:**
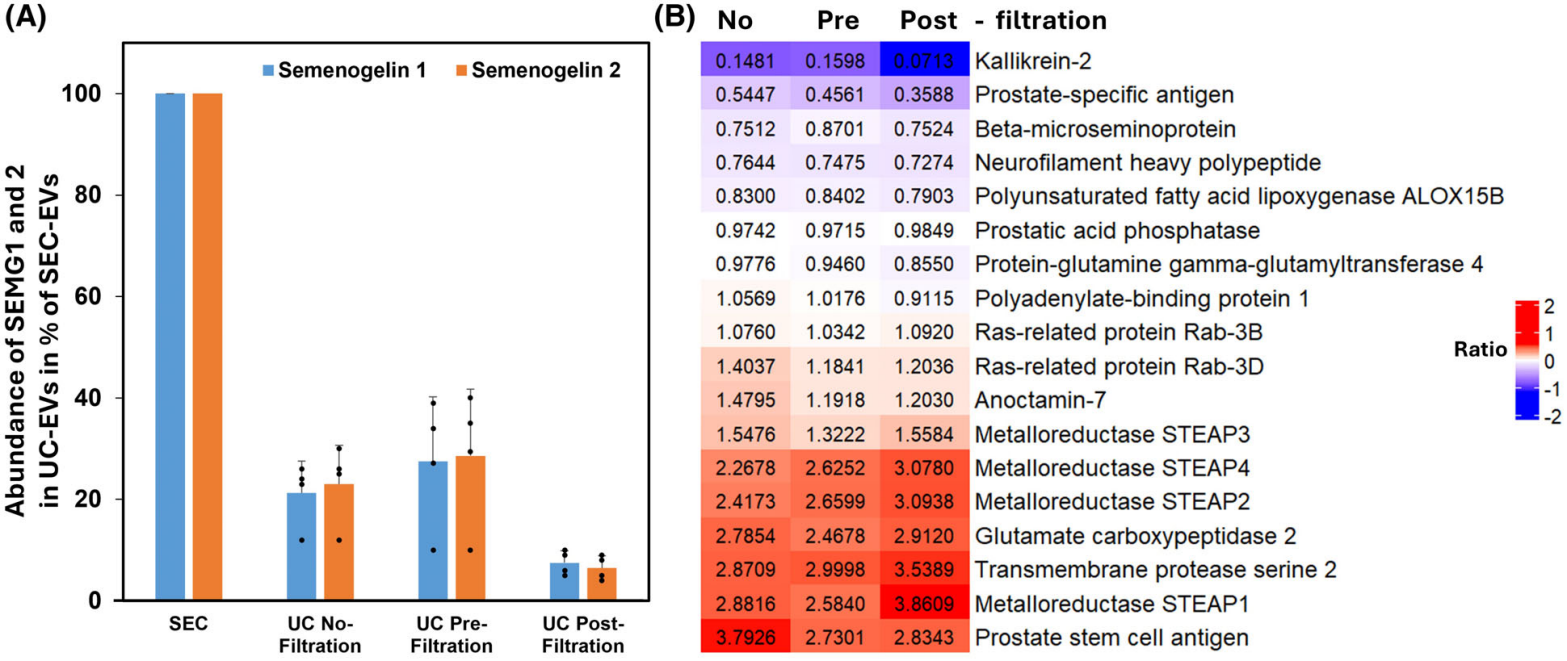
Comparison of the abundance of semenogelins and prostate‐related proteins in seminal small EV samples prepared by UC and SEC. (A) The figure shows the label‐free quantification abundance of semenogelin 1 (SEMG1) and semenogelin 2 (SEMG2) in EV samples prepared by three UC protocols in percentage to its abundance in EV samples prepared by SEC. Error bars represent the standard deviation (SD) of two donors, with samples for each protocol obtained in duplicate for each donor (*n* = 4). Individual data points are shown in the figure. (B) Heatmap showing the protein abundance ratio of selected prostate‐related proteins in seminal EV samples prepared by three UC protocols compared to SEC. For ease of visualization, the color scale shows the log_10_‐transformed ratios. Proteins are ordered by ratio. The ratio values are also included in the heatmaps. Order of columns from left to right: No, UC no‐filtration vs SEC; Pre, UC prefiltration vs SEC; Post, UC postfiltration vs SEC. EVs, extracellular vesicles; SEC, size‐exclusion chromatography; STEAP, six‐transmembrane epithelial antigen of the prostate; Rab, Ras‐related small GTPase; UC, ultracentrifugation.

Since PCa is a main male cancer worldwide [43], we specifically investigated the levels of several prostate‐related proteins in seminal fluid small EV samples prepared by SEC and UC. As shown in Fig. 7B, particularly PSA (KLK3) and KLK2 had lower abundance in UC‐EVs compared to SEC‐EVs. Other prostate‐related proteins showed similar levels in both SEC and UC prepared seminal EVs, such as PAP, TGM4, and Rab3, thus suggesting that both protocols may be used. Finally, other proteins, such as STEAP proteins, FOLH1, TMPRSS2, and PSCA, were enriched in UC seminal EVs compared to SEC‐EVs.

### 3.4 Proteomic analysis of seminal small EVs prepared by UC with and without filtration

A 200 nm filtration step in the UC protocol was added to assess whether it reduced the co‐isolation of large protein complexes in EV preparations, as previously demonstrated for urinary EVs and uromodulin [42]. To characterize the effect of filtration, the ratios between the abundance of proteins in EVs prepared using a filtration step and without a filtration step were calculated. As shown in File S1, Table 3, the effect of filtration before EV pelleting was minimal (three proteins considering a fold chain of 1.5). However, more changes were observed when the filtration step was performed after EV pelleting. In this case, it was found than 36 proteins were decreased, and two proteins were increased (fold change 1.5) in EVs prepared with the UC postfiltration protocol compared to UC with no filtration (File S1, Table S4, Fig. 8). The proteins that were less abundant in EVs prepared by UC postfiltration compared to unfiltrated samples were mainly secreted proteins, that is, semenogelins, laminins, clusterin, fibronectin, gastricsin, beta‐hexosaminidase subunit alpha, and sphingomyelin phosphodiesterase. Interestingly, many these proteins were also found to be less abundant in EVs prepared by UC with no filtration compared to SEC‐EVs (Fig. 6). This indicates that even if the UC protocol removes these proteins from the EV samples more efficiently than SEC, they can be further depleted by filtrating the samples after pelleting the EVs. Whether this is due to the filtration step perturbing the EV corona or removing potential aggregates of these proteins, is unclear.

**Fig. 8:**
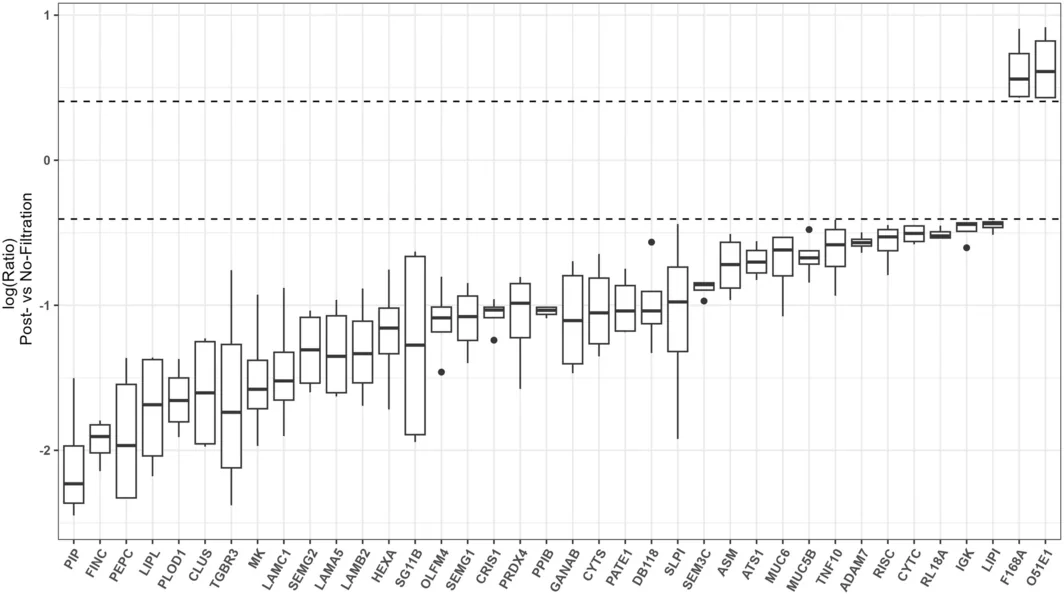
Proteins differentially abundant in seminal small EVs prepared by UC No‐filtration compared to UC Post‐filtration. The boxplots show the protein abundance ratio between UC Post‐filtration compared to UC No‐filtration. For this analysis, four ratios were calculated based on the samples available. Proteins showing increased or decreased abundance by a ratio larger than 1.5 in all the four samples available per protocol were selected. The four ratios were used to calculate the mean ratio. Only proteins identified in all the samples were considered. The box plots represent the interquartile range (IQR). The whiskers extend to 1.5 times the IQR above and below the box. EVs, extracellular vesicles; UC, ultracentrifugation.

## 4 Discussion

The comparison of four different methods to prepare EVs from seminal plasma show that SEC and UC are enriched with different proteins, thus suggesting that they include different EV populations. The EV population obtained by UC has a higher abundance of commonly EV‐related proteins compared to EVs prepared by SEC, whereas EVs prepared by SEC are enriched in ER and ribosomal proteins. In addition, several PCa biomarkers were differentially enriched in SEC and UC preparations. Finally, we observed that UC samples contained less non‐vesicular proteins and/or EV corona proteins.

When working with EVs and liquid biopsies, abundant proteins present in biofluids may compromise the analysis of EV‐based biomarkers [32, 44, 45]. These proteins can form large aggregates that co‐isolate with EVs and/or bind to EVs and contribute to the EV corona [46, 47, 48]. Understanding the nature of these proteins and their impact on biomarker detection is therefore essential for the clinical use of EVs. Our results indicate that the abundance of several potential non‐vesicular associated proteins and/or corona proteins is lower in EVs prepared by UC compared to SEC. Examples of these proteins are semenogelins, immunoglobulins, fibronectin, albumin, antileukoproteinase, beta‐2‐microglobulin, gastricsin, KLK2, mucins, prolactin‐inducible protein, nephronectin, olfactomedin‐4, and apoliproteins [41, 46]. In terms of the proteins that form the EV corona, it has been shown that both UC and SEC can affect them to some extent [32, 48]. If we consider that the secreted proteins found in our EV preparations are part of the EV corona, we could say that UC removes more efficiently the EV corona, which seems to be good for the detection of EV‐membrane biomarkers [32]. Moreover, a filtration step after UC pelleting, additionally removes these proteins from EV preparations. It is not clear why this is the case. Protein aggregates could be retained in the filters, as has been shown for uromodulin [42], but it could also affect the EV corona.

Both removal of the corona and a better removal of aggregates by UC would result in a lower protein amount in UC‐EVs compared to SEC‐EVs, and this is what we observed. Moreover, western blot analysis showed that the levels of EV‐associated markers (CD81 and CD9) were not higher in SEC‐EVs than UC‐EVs, thus suggesting that SEC isolation does not increase the EV yield. This finding is consistent with previous studies showing that EVs isolated by SEC often exhibit lower abundance of canonical EV markers, such as CD9, CD63, and CD81, compared to those isolated by UC or precipitation‐based methods [49, 50].

In the UC protocol, we investigated whether incorporating a 0.2 μm filtration step, either before or after pelleting EVs, could improve the purity of the samples. Our results showed that, compared to nonfiltrated samples, the use of a 0.2 μm filtration step after EV pelleting reduced the total amount of protein and the levels of several nonvesicular proteins and/or corona proteins more effectively than before EV pelleting. It is not clear why this is the case, but it is possible that after UC, the nonvesicular proteins and/or EVs in the pellet are easily retained in the filter [51]. In fact, western blot analysis showed that the levels of CD9 were lower in these samples. Therefore, including this filtration step may require a more detailed evaluation in terms of EV yield versus purity.

Based on the presence and abundance of numerous PCa related proteins, seminal EVs appear to be a good source for PCa biomarkers. However, we observed that the distribution of several prostate‐related proteins differed between SEC‐EV and UC‐EV preparations. SEC‐EVs were primarily enriched in prostate‐derived proteins considered to be soluble and/or part of the corona, such as KLK3 (PSA) and KLK2. In contrast, UC‐EV preparations were enriched in prostate‐related membrane proteins, including PSCA, STEAP1, and STEAP2. Based on this, it is important to identify which protocol fits better for the biomarker to be investigated. We would also like to point out that urinary EVs have often been used in PCa biomarker research [52]. However, since prostatic fluid is a central component of seminal fluid, it can be expected that PCa biomarkers are likely to be more abundant in seminal EVs than in urinary EVs, potentially making them easier to detect and quantify. Still, semen collection can be challenging in PCa patients, particularly due to advanced age or clinical conditions. When semen collection is not feasible, alternative approaches to enrich prostate‐derived EVs in urine samples may be used, such as collecting urine after a prostatic massage or specifically immunoisolating prostate‐related EVs from urine [53].

Finally, our results suggest that EVs prepared by SEC and by UC are enriched in different EV populations. EVs prepared by UC were enriched by proteins commonly associated with small EVs [54], such as tetraspanins, alix, tsg101, flotillins, and Rabs. In addition, all ESCRT proteins, known for their role in the formation of intraluminal vesicles from multivesicular bodies [55], as well as other vesicular transport‐related proteins such as SNAREs, sorting nexins, and guanine nucleotide‐binding proteins, were enriched in these EV samples. In contrast, EVs prepared by SEC were enriched in ribosomal proteins and ER‐related proteins. This is interesting considering the recent discovery of a subpopulation of small EVs originating from the ER [56, 57, 58, 59]. In addition, several enzymes related to lysosomes/secretory granules were also enriched in EVs isolated by SEC (Beta‐hexosaminidase, cathepsin B and D, acid ceramidase, pancreatic glycoprotein 2, and alpha‐glucosidase). This suggests that proteins associated to secretory granules may to some extent end up in this fraction. The reasons for the differential enrichment in EV populations by UC or SEC remain unclear, but they may relate to differences in the size and/or density of seminal EVs. Indeed, a study using sucrose gradient centrifugation identified two distinct seminal EV populations following sucrose gradient centrifugation [23]. Moreover, it has been shown that small EVs originating from endosomes and from the ER exhibit some differences in density and size [57].

## 5 Conclusion

In conclusion, our findings demonstrate that the choice of preparation method strongly influences the EV population isolated from seminal fluid, as well as the presence of nonvesicular proteins and potential corona proteins in the samples. Further validation using complementary methods and larger cohorts would be beneficial, as these parameters may have a significant impact on the detection and interpretation of EV‐associated cancer biomarkers and should therefore be carefully considered in future studies.

## Conflict of interest

The authors declare no conflict of interest.

## Author contributions

The study was designed by AL, MBR, and M‐BT. ET was responsible for sample collection and protocol optimization, in collaboration with BM‐G and KS. Data analyses were performed by ET and EB‐C, with guidance and feedback from AL, TAN, MBR, MKA, and M‐BT. ET and AL drafted the manuscript with input from all authors. All authors have read and approved the final version of the manuscript.

## Supporting information


**File S1.** Excel file containing four tables.

**Figure S1.** Analysis of cleared seminal fluid after SEC by dynamic light scattering.
**Figure S2.** Principal component analysis of SEC‐EVs and UC‐EVs (No Filtration, Pre‐Filtration and Post‐ Filtration) in donor 1 and donor 2.
**Table S1.** Normalized LFQ values of the proteins identified in seminal small EVs.
**Table S2.** EV‐protein abundance ratios between the three UC protocols (No filtration, Pre‐Filtration, and Post‐Filtration) and SEC.
**Table S3.** EV‐protein abundance ratios between the UC Pre‐Filtration protocol and UC No filtration.
**Table S4.** EV‐protein abundance ratios between the UC Post‐Filtration protocol and the UC No Filtration.

## Data Availability

All the data and materials are presented as part of this manuscript and the Supporting Information. The mass spectrometry proteomics data have been deposited in the ProteomeXchange Consortium via the PRIDE partner repository with the dataset identifier PXD069636.
