# Supplementary material for: Optimization of seminal extracellular vesicle isolation protocols for cancer biomarker analysis using proteomic profiling
Source: Mol Oncol. 2026 Oct 22:10.1002/1878-0261.70345. Online ahead of print. doi: 10.1002/1878-0261.70345 (PMC13688550; doi:10.1002/1878-0261.70345)
Supplement: Supplementary file 1 [file MOL2-9999-0-s002.docx]

## **Supporting Information**

**Supporting file**

**File S1.** Excel file containing four tables 1.

**Table 1. Normalized LFQ values of the proteins identified in seminal small EVs.** In total, 14 samples were analyzed: Seven samples from donor 1 and seven samples from donor 2, including EVs isolated by three UC-based protocols performed in duplicate and EVs isolated by SEC as a single sample. LFQ: Label-Free Quantification. EVs: Extracellular vesicles. UC: Ultracentrifugation. SEC: Size-Exclusion Chromatography.

**Table 2. EV-protein abundance ratios between the three UC protocols (No filtration, Pre-Filtration, and Post-Filtration) and SEC.** For this analysis, four ratios were calculated based on the four samples available for each UC-protocol. Proteins showing increased or decreased abundance by a ratio larger than 1.5 compared to SEC in all the four samples available per UC protocol were selected. The four ratios were used to calculate the mean ratio and standard deviation. Only proteins identified in all the samples were considered for the analysis. UC: Ultracentrifugation. SEC: Size-Exclusion Chromatography. EV: Extracellular vesicle.

**Table 3. EV-protein abundance ratios between the UC Pre-Filtration protocol and UC No filtration.** Four ratios were calculated based on the samples available per protocol. Proteins showing increased or decreased abundance by a ratio larger than 1.5 were selected. The four ratios were used to calculate the mean ratio and standard deviation. Only proteins identified in all 14 samples were considered for the analysis. UC: Ultracentrifugation. EV: Extracellular vesicle.

**Table 4. EV-protein abundance ratios between the UC Post-Filtration protocol and the UC No Filtration.** Four ratios were calculated based on the samples available per protocol. Proteins showing increased or decreased abundance by a ratio larger than 1.5 were selected. The four ratios were used to calculate the mean ratio and standard deviation. Only proteins identified in all the samples were considered for the analysis. UC: Ultracentrifugation. EV: Extracellular vesicle

**Supporting figures**

**Figure S1. Analysis of cleared seminal fluid after SEC by dynamic light scattering.** The count rate of a similar volume of each SEC fraction was measured by dynamic light scattering in a Zetasizer instrument. The figure shows the results of a representative donor; similar results were consistently observed across three donors. SEC: Size-Exclusion Chromatography. F: Fraction. Kpcs: kilo counts per second.

**Figure S2.** Principal component analysis of SEC-EVs and UC-EVs (No Filtration, Pre-Filtration and Post- Filtration) in donor 1 and donor 2. SEC: Size-Exclusion Chromatography. UC: Ultracentrifugation. EVs: Extracellular vesicles.

**Figure S1**

**Figure S2**
